# 

**Published:** 1904-08-20

**Authors:** 


### NOTICE TO CORRESPONDENTS.

All MSS., Letters, Books for Review, and other matters intended for the Editor should be addressed The Editor, "The Hospital," The Hospital Buildings, 28 & 29 Southampton Street, Strand, W.C.

The Editor cannot undertake to return rejected MS., even when accompanied by stamped directed envelope.

**Notice to Advertisers and Subscribers.**

All Advertisements, Orders for Copies of The Hospital, and Business Communications should be addressed to **THE MANAGER (Not to the Editor)**, The Hospital, 28 & 29 Southampton Street, Strand, London, W.C.

The Cost of Subscription, post free, is as follows:—Per week, 3½d.; per quarter, 3s. 3d.; per half-year, 6s. 6d.; per annum, 13s. Foreign and Colonial:—Per annum, 19s. 6d., payable, in all cases, in advance.

**NOTICE.—Vol. XXXV. of "The Hospital" (October 1903 to March 1904), in handsome cloth binding, is now on sale at the office of this Journal, price 10s. 6d. Cases for binding the Numbers contained in this Volume 2s. Index to Vol. XXXV., 3½d., post free.**

**The Monthly part for August is now ready. Price 1s., by post, 1s. 3d.**

### BOOKS RECEIVED.

Baillière, Tindall and Cox.

"The Cancer Problem in a Nutshell." By R. Bell, M.D.

John Dicks.

"The Last of the Mohicans."
"Rattlin the Reefer."
"Nights at Sea."

### MEDICAL APPOINTMENTS.

**R. G. Archibald**, M.B., Ch.B.Edin., Assistant Resident Medical Officer at the St. Mary's Hospital for Sick Children, Plaistow. **Arthur Bernard Cridland**, F.R.C.S.Edin., L.R.C.P. Lond., Honorary Assistant Surgeon to the Wolverhampton and Midland Counties Eye Infirmary, and Honorary Ophthalmic Surgeon to the Royal Orphanage, Wolverhampton. **J. F. Halls Dally**, M.A., M.B., B.C.Cantab., M.R.C.S.Eng., L.R.C.P.Lond., Senior Resident Medical Officer to the Royal National Hospital for Consumption and Diseases of the Chest, Ventnor, I.W. **J. M. Dupont**, M.B., Ch.B.Edin., an Assistant Resident Medical Officer to the Royal National Hospital for Consumption and Diseases of the Chest, Ventnor, I.W. **W. Marcus Killen**, B.A., M.D., M.Ch., Acting Surgeon to Benn Ulster Eye, Ear, and Throat Hospital, Belfast. **Thomas David Lister**, M.D.Lond., M.R.C.P. Lond., Honorary Consulting Physician to the Benevolent Fund of the National Union of Teachers. **Henry MacCormac**, M.B., Ch.B.Edin., Assistant Resident Medical Officer to the Royal National Hospital for Consumption and Diseases of the Chest, Ventnor, I.W. **D. G. MacDonald**, M.B., C.M., D.P.H.Aberd., Certifying Factory Surgeon for the Stornoway District. **Ewen J. Maclean**, M.D., M.R.C.P.Lond., F.R.S.Edin., Lecturer in Midwifery (under the Midwives Act) to the University College of South Wales and Monmouthshire, Cardiff. **C. A. Robinson**, B.C., C.M.Cantab., Medical Officer of Health for the Godstone Rural District. **Hugh F. Sheldon**, M.R.C.S., L.R.C.P.Lond., Civil Surgeon, Military Hospital, Potchefstroom, South Africa. **John H. Sheldon**, M.B.Lond., etc., Medical Officer to the Globe and Phœnix and Gaika Gold Mining Companies, Sebakine, Rhodesia. **P. R. Tarbett**, M.R.C.S.Eng., L.R.C.P., Certifying Factory Surgeon for the Stevenage District, co. Hereford.

### "The Hospital" Institutional Library.

"Hospitals and Asylums of the World." 4 Vols., with a Portfolio of Plans, complete £12 12s.

"Burdett's Hospitals and Charities, 1904." 5s. net.

"Burdett's Official Nursing Directory." 8s. net.

"Cottage Hospitals, General, Fever, and Convalescent." 10s. 6d.

"Uniform System of Accounts for Hospitals and Public Institutions." New Edition. 4s. net.

"Hospital Expenditure: The Commissariat." 2s. 6d.

"A Legal Handbook for the Use of Hospital Authorities." 2s. 6d.

"The Cottage Hospital Case Book or Register of Patients." 10s. and 12s. 6d.

All these are published by the Scientific Press, Ltd., and may be obtained through any bookseller, or direct from the publishers, 28 and 29 Southampton Street, Strand, London, W.C.

### The L.O.S.

NURSES ENTERING FOR THE NEXT EXAMINATION OF THE LONDON OBSTETRICAL SOCIETY

**Should obtain a Copy of**

### A COMPLETE HAND-BOOK OF MIDWIFERY.

**By J. K. WATSON, M.D.,**
Author of "A Handbook for Nurses."

Contains over 350 pp., Illustrated with 143 excellent Illustrations, bound in cloth.

London Hospital Journal says :—"In this volume is maintained the high standard of utility set in his (Dr. Watson's) Handbook for Nurses. . . . **Will be very useful to candidates for the L.O.S.**"

Price

**6/-** net;

**6/4** post free.

The Nurses' Journal says :—"This is a very complete and exhaustive treatise on the subject, and we feel that **we can with every confidence recommend it as a safe and reliable guide.**"

**FULL PROSPECTUS POST FREE ON APPLICATION.**

***NEW EDITION NOW READY.***

THE

### NURSE'S PRONOUNCING DICTIONARY

### Of Medical Terms and Nursing Treatment.

**Compiled by HONNOR MORTEN.**

**FIFTH EDITION, with Phonetic Pronunciations,**

**Revised and Edited by MARY I. BURDETT,**
Member of Guy's Hospital Past and Present Nurses' League.

Price
**2/-**
post free.

THE value of this well-known Dictionary has been greatly enhanced by the many new and important additions to the text, **while the fact that the phonetic pronunciation is now appended to the majority of the words will add immeasurably to the practical utility of this already indispensable Companion to Nurses.**

Price
**2/-**
post free.

SUITABLE SIZE FOR THE APRON POCKET.

162 pp., cloth, **2/-** post free; or in handsome leather gilt, **2/6** net, **2/8** post free.

**New Complete Catalogue of Nursing Manuals Post Free on Application.**

